# TIGAR promotes osteogenic differentiation and ameliorates glucocorticoid-induced osteoporosis via autophagy-Nrf2-ROS axis

**DOI:** 10.1016/j.gendis.2025.101735

**Published:** 2025-06-26

**Authors:** Dingmei Zhang, Feng Ding, Yizhong Wang, Jie Cheng, Jiaxing Zhu, Shiyu Liu, Xin Wang, Zheng-Hong Qin, Lili Ren

**Affiliations:** a State Key Laboratory of Oral & Maxillofacial Reconstruction and Regeneration, National Clinical Research Center for Oral Diseases, Shaanxi Key Laboratory of Stomatology, Department of Oral Biology & Clinic of Oral Rare Diseases and Genetic Disease, School of Stomatology, The Fourth Military Medical University, Xi'an, Shaanxi 710032, China; b Department of Orthopaedic Surgery, Affiliated Hospital of Zunyi Medical University, Zunyi, Guizhou 563003, China; c State Key Laboratory of Oral and Maxillofacial Reconstruction and Regeneration, National Clinical Research Center for Oral Diseases, Shaanxi International Joint Research Center for Oral Diseases, Center for Tissue Engineering, School of Stomatology, The Fourth Military Medical University, Xi'an, Shaanxi 710032, China; d Institute of Health Technology, Global Institute of Software Technology, Qingshan Road, Suzhou Science & Technology Tower, Hi-Tech Area, Suzhou, Jiangsu 215163, China; e Department of Sports Medicine, Honghui Hospital of Xi'an Jiaotong University, Xi'an, Shaanxi 710032, China; f State Key Laboratory of Military Stomatology and National Clinical Research Center for Oral Diseases and Shaanxi International Joint Research Center for Oral Diseases, Department of Radiology, School of Stomatology, The Fourth Military Medical University, Xi'an, Shaanxi 710032, China

**Keywords:** Autophagy, Nrf2, Osteoporosis, Oxidative stress, TIGAR

## Abstract

Glucocorticoid-induced osteoporosis (GIOP) is a public health problem that needs urgently to be resolved, and oxidative stress is closely related to osteogenic impairment. TP53-induced glycolysis and apoptosis regulator (TIGAR) contributes to the occurrence and development of various diseases by reducing reactive oxygen species (ROS). However, it is unknown whether and how TIGAR plays a regulatory role in GIOP. The aim of the present study is to investigate the role of TIGAR in osteogenic differentiation and the underlying molecular mechanism. We explored the protective role and mechanism of TIGAR on osteogenic differentiation and GIOP by using the TIGAR overexpression plasmid and siRNA *in vitro*, and by constructing systemic TIGAR overexpression (TG-TIGAR) mice *in vivo*, respectively. In conclusion, our study clarified that TIGAR promotes osteogenic differentiation and improves GIOP by upregulating autophagy-nuclear factor erythroid-2 related factor (Nrf2)-ROS pathway, suggesting that TIGRA may be a potential therapeutic target for GIOP treatment.

## Introduction

Osteoporosis (OP) is a systemic bone disease that is characterized by the decrease of bone density and quality, the damage of bone microstructure, and the increase of bone fragility. Glucocorticoids (GC) are widely used in clinically owing to their extensive pharmacological effects, and it is estimated that 1%–2% of people worldwide receive long-term GC therapy.^1^ However, long-term use of GC can damage bone mass and quality and is the most common secondary cause of osteoporosis and osteoporotic fractures. Glucocorticoid-induced osteoporosis (GIOP) is a public health problem that has attracted substantial attention because it not only has a significant adverse impact on patient quality of life, but also imposes an economic burden on families and society.^2^ The prevalence of GIOP among patients with rheumatology in Africa was 47.7%,^3^ and secondary osteoporosis and fractures may occur in up to 30–50% of patients treated with GC.^4^^,^^5^ GC has an incremental cost of $3201 per fracture and $888 per osteoporosis at cumulative doses greater than 1800 mg, according to a recent assessment of adverse events.^6^ There have been several new drugs developed for treating osteoporosis, but their use is limited. For example, long-term use of antiresorption drugs, such as bisphosphonates, increases bone fragility and osteonecrosis of the jaw due to the interrupted coupling of bone resorption and bone formation.^7^ And teriparatide, which promotes bone formation, is contraindicated in a variety of situations (such as hypercalcemia and lactation).^1^ Therefore, it is vital to understand the new mechanisms underlying GIOP in order to develop new therapeutics and preventative strategies.

The potential mechanism of GIOP has not been identified, and oxidative stress is one of the underlying mechanisms of GIOP.^8^^,^^9^ Overproduction of reactive oxygen species (ROS) not only induces defective differentiation and maturation of osteoblasts, but also increases their apoptosis and reduces their ability to form bones.^10^^,^^11^ The activity of osteoclasts, osteoblasts, bone mesenchymal stem cells (BMSCs), and osteocytes participated in the bone formation of matrix can be affected apoptosis.^12^^,^^13^ GC-induced dysfunction and osteoblast apoptosis are considered crucial factors in GIOP,^14^^,^^15^ and mice treated with dexamethasone (Dex) showed increased ROS levels with subsequent induction of osteoblast apoptosis and decreased bone mass.^16^^,^^17^ Furthermore, osteoblast apoptosis induced by injury of the antioxidant defense system (such as reduced reactivity of antioxidant enzymes) plays a leading role in the progression of GIOP,^16^^,^^18^ and activation of antioxidant pathways protects osteoblasts from oxidative stress.^19^^,^^20^ These studies suggest that oxidative stress has an important regulatory effect on osteoporosis.

TP53-induced glycolysis and apoptosis regulator (TIGAR), a protein with bisphosphatase activity that contributes to ROS limitation by activating the oxidative pentose phosphate pathway (PPP) to produce NADPH for antioxidant defense,^21^^,^^22^ and thus protect ROS-sensitive apoptotic responses.^23^ Although initially identified as a target of P53, the regulation of TIGAR expression and some biological functions are generally independent of P53.^24^ In addition to maintaining energy metabolism, TIGAR also regulates stem cell differentiation and autophagy, and promotes cell survival. Consequently, it plays a role in a variety of diseases, including cancer, stoke, and heart failure.^25^ In recent studies, TIGAR has been shown to be effective in preventing degenerative lumbar disc herniation by enhancing the ratios between NADPH/NADP^+^ and GSH/GSSG in the nucleus pulposus cells.^24^^,^^26^ However, whether TIGAR play a regulatory role in GIOP and the potential mechanisms remain unclear.

As a key regulator of oxidative stress and antioxidant defenses, nuclear factor erythroid-2 related factor (Nrf2) plays a crucial role in bone-related diseases such as osteoporosis.^27^^,^^28^ Nrf2 can move to the nucleus under oxidative stress and enhances the expression of antioxidant enzymes like superoxide dismutase (SOD), glutathione peroxidase (GPx), and catalase (CAT).^29^^,^^30^ The activation of various antioxidant enzymes protects bone tissues from oxidative stress-induced damage.^28^ Previous studies have indicated that Nrf2 prevents Dex-induced oxidative stress and apoptosis to prevent osteoblast damage,^31^ and Nrf2-deficient mice exhibited decreased bone mineral density (BMD) and cortical bone area in the vertebrae.^32^ It appears that Nrf2 and TIGAR may coordinate in some disease processes to directly stimulate PPP and other NADPH-generating enzymes for redox homeostasis.^33^, ^34^, ^35^ Furthermore, TIGAR was reported to activate Nrf2 by inducing autophagy to degrade kelch-associated protein 1 (Keap1) in neurons with prolonged ischemia.^36^ There is still a question as to whether and how TIGAR regulates Nrf2 in osteogenic differentiation to reduce oxidative stress.

Our study examined the role and mechanisms of TIGAR in osteogenic differentiation in response to Dex treatment and GIOP. Our results indicate that TIGAR protects against oxidative stress by promoting the antioxidant capacity of osteoblasts by regulating autophagy-dependent Nrf2 nuclear translocation and enhancing osteogenic differentiation, thus alleviating the development of GIOP, suggesting that TIGAR overexpression may be a therapeutic strategy of GIOP. In summary, these findings shed light on the pathogenesis of GIOP and potential prevention strategies.

## Materials and methods

### Materials and reagents

The Dex injection was provided by Sinopharm Group Rongsheng Pharmaceutical Co., Ltd. (Henan, China), and *in vitro* Dex was purchased from Sigma (St. Louis, MO, USA, D4902). Beyotime Biotechnology (Shanghai, China) provided N-Acetyl-l-cysteine (NAC, S0077), RIPA lysis buffer, and bicinchoninic acid (BCA) protein assay kit. It was obtained from GenePharma (Suzhou, China) the TIGAR overexpression plasmid as well as siRNA oligonucleotides. Enzyme-linked immunosorbent assay (ELISA) kits provided by Jiangsu Meimian Industrial Co., Ltd (Jiangsu, China) was used to test mouse C-terminal cross-linked peptide of type I collagen (CTX-I) (CAS No. 24313) and mouse N-terminal propeptide of type I collagen (PINP) (CAS No. 19685) were purchased from. Trizol reagent, reverse transcription kits and TB Green® Premix Ex Taq™ II were purchased from TaKaRa (Tokyo, Japan). α-Minimum essential medium (α-MEM) and fetal bovine serum (FBS) were obtained from Gibco (Grand Island, NY, USA). All the other reagents were of analytical grade.

### Animals and treatment

As the rate of osteoporosis in women over 50 years of age is four times that of men in the same age group,^37^ we selected female animals that are more prone to osteoporosis in this study. We obtained female C57BL/6 mice aged 8 weeks from the animal center of the Fourth Military Medical University (Xi'an, China). The TIGAR-transgenic (TG-TIGAR) and matched WT mice were kindly provided by Professor Qin (Soochow University, China) and the strategy of mice generation was described previously.^38^^,^^39^ Mice were kept in pathogen-free cages and fed a standard rodent diet and water under12-h light/12-h darks cycles. All experimental procedures were approved by the Ethical Committee of the Fourth Military Medical University following the International Association of Laboratory Animal Care Assessment and Certification (AAALAC International). The GIOP animal model was conducted with reference to the previous literature.^40^^,^^41^ The mice were randomly divided into three groups (*n* = 5 per group): control, Dex-L, and Dex-H. Mice in the Dex-L group were injected with Dex at a dose of 2 mg/kg body weight and the Dex-H group was 5 mg/kg by intramuscular injection twice a week. Normal saline was given to the control group in equal volume. And in the experiments with TIGAR transgenic mice, Dex was administered at a dose of 5 mg/kg twice a week. After 8 weeks of treatment, the femurs were subjected to micro-CT and histomorphometric analyses. Blood was collected and centrifuged at 3000× *g* for 15 min at 4 °C to separate serum from plasma, which was immediately analyzed or refrigerated at −20 °C.

### Micro-CT analysis

A Skyscan 1276 micro-CT imaging system (Skyscan, Kontich, Belgium) with an 8 μm spatial resolution (X-ray source 55 kV/200 μA) was used for the micro-CT examination. NRecon 1.6 and CTAn 1.8 were used for volume reconstruction. Bone remodeling was assessed by defining the volume of interest as the cylindrical space covering cancellous bone. It was calculated within the delimited volume of interest the trabecular bone volume (BV/TV, %), the number of trabecular bones (Tb.N, mm), and trabecular separation (Tb.Sp, mm).

### ELISA

Commercial ELISA kits (Jiangsu Meimian Industrial Co., Ltd.) were used to measure serum PINP and CTX-I concentrations.

### Immunofluorescence staining

To detect TIGAR expression in the femur, the femur tissue was fixed in 4% paraformaldehyde solution for 48 h and treated according to the frozen section technique. Following washing and permeation with 0.1% Triton X-100 in PBS for 20 min at room temperature (25 °C), the sections were blocked with 5% bovine serum albumin in PBS for 1 h. Incubation of TIGAR primary antibody (1:100; Santa Cruz, sc-166290) at 4 °C overnight was followed by incubation of secondary fluorescent antibody (1:100; Bioss Inc., Beijing, China). 4′,6-diamidino-2-phenylindole (DAPI) was next incubated on the sections for 5 min at room temperature and observed using a confocal microscope (Nikon, Japan). For cell immunofluorescence, in a similar manner to tissue sections, cells fixed on coverslips in 4% paraformaldehyde were subjected to similar procedures. Primary antibodies LC3 (1:100, Cell Signaling Technology, #12741), Keap1(1:100, Zen-bio, R26935) and Nrf2 (1:100, Zen-Bio, 380773) were incubated overnight at 4 °C. ImageJ software (NIH Bethesda, MD, USA) was used to analyze the captured images and determine the fluorescence intensity in five different fields of view.

### Cell culture and treatments

BMSCs were harvested from 3-week-old mice and grown in α-MEM containing 10% FBS, 100 units/mL penicillin, and 100 mg/mL streptomycin (Gibco). The identification of BMSCs was carried out by analyzing the cell surface positive markers CD105 and Sca1, as well as the negative markers CD34 and CD45 using flow cytometry (Fig. S1). After euthanization of the mice, the bone marrow of tibiae and femurs was rinsed with α-MEM. A 40-μm cell filter was used for the filtering process and cells were cultured in 5% CO_2_ for 4 days at 37 °C at a density of 1 × 10^5^ per cm^2^. BMSCs from passages 2 to 4 were used for osteogenic differentiation experiments. Osteogenic differentiation was induced by adding osteogenic induction medium (10% FBS in α-MEM with 25 mg/mL Vit C and 5 mM β-glycerophosphate) to 6-well cell culture plates, with medium changes every 3 days. For cell treatment, with reference to previous studies,^42^^,^^43^ Dex was administered at different concentrations (0, 500, 1000 and 2000 nM) for 48 h to explore its effects on osteoblast and TIGAR expression. In subsequent experiments, Dex 2000 nM was added to the cells for 48 h, unless otherwise specified. Nrf2 inhibitor (10 μM) (ML385, TargetMol Chemicals Inc., USA, CAS846557-71-9) and chloroquine (CQ, TargetMol Chemicals Inc., CAS54-05-7) in 20 μg/mL were administered to cells to explore the underlying mechanisms. To evaluate autophagic flux, BMSCs were transfected with the mCherry-GFP-LC3 plasmid (GenePharma) using Advanced DNA/RNA Transfection Reagent (Zetalife, USA, AD600025).

### Gene overexpression and knockdown assay

GenePharma designed and synthesized siRNA duplexes specific to TIGAR and TIGAR-overexpression plasmids. BMSCs were transiently transfected with TIGAR siRNA or a plasmid overexpressing TIGAR according to the manufacturer's protocol using advanced DNA/RNA transfection reagent. A negative control (NC) plasmid or siRNA was used as the control. Within 24 h of transfection, BMSCs were provided with fresh medium and administered Dex and/or ML385/CQ for 48 h. Transfection efficiency was determined by quantitative real-time polymerase chain reaction (qRT-PCR) and Western blot.

### Cell viability assay

Cell viability of BMSCs after Dex treatment was detected by sulforhodamine B (SRB) cytotoxicity assay kit (Yeasen Biotechnology Co., Ltd., Shanghai, China, 40205ES76). Briefly, 1 × 10^4^ BMSCs were plated in 96-well plates at a density of and treated with Dex for 24, 48, and 72 h. Next, cells were rinsed with PBS solution after removing culture medium and fixed for 1 h at 4 °C. Then, the cells were washed and incubated with SRB for 15 min at room temperature. The excess dye was removed, and protein-bound dye was dissolved with dissolving solution. A microplate reader (Bio-Rad, Hercules, CA, USA) was used to measure the absorbance at 515 nm. Cell viability was calculated as (average absorbance of the Dex treatment group/average absorbance of the control group) × 100%.

### ALP staining and Alizarin red S staining

Osteoblasts differentiated from BMSCs for 9 d in 6-well plates were fixed for 20 min at room temperature in 4% paraformaldehyde and incubated with BCIP/NBT solution (Beyotime, C3206) for 60 min according to the manufacturer's instructions. The ALP-positive area was analyzed using the ImageJ software and is shown as a percentage of the ALP-positive area over the total area. For Alizarin Red S staining, after osteogenic induction for 2 weeks, the cells were fixed, rinsed, and incubated with Alizarin Red S (Beyotime, C0148S). Mineral deposits containing differentiated cells were stained with Alizarin Red S solution. Images were captured using a digital camera. Alizarin red-positive areas were analyzed using ImageJ software and are shown as a percentage of the Alizarin Red-positive area over the total area.

### Measurement of intracellular ROS levels

The ROS-specific fluorescent probe dihydroethidium (DHE, Beyotime, S0063) was used to measure total intracellular ROS levels. BMSCs (1 × 10^5^ cells) were cultured overnight in 12-well plates and treated with Dex after TIGAR overexpression or knockdown. Then, cells were incubated with fresh medium containing 5 mM DHE at 37 °C for 30 min. A fluorescence microscope was used to observed the intensity of fluorescence. Optical density was measured in five different fields of view for each sample to assess staining intensity.

### Determination of cell apoptosis

Annexin V-FITC/propidium iodide (PI) staining kit (Beyotime, C1062) was used to determine the apoptosis of BMSCs. Briefly, following digestion with trypsin 0.25%, BMSCs were centrifuged (1000× *g* for 5 min), stained with Annexin V-FITC for 10 min, and PI for 5 min, and then quantified with flow cytometry (FACSCaliber; Becton Dickinson, Heidelberg, Germany).

### Measurement of NADPH/NADP^+^ ratio and GSH/GSSG content

Intracellular NADPH/NADP^+^ levels were detected by NADP^+^/NADPH assay kit (Beyotime, S0179), and GSH and GSSG contents were determined using the GSH and GSSG assay kit (Beyotime, S0053) according to the manufacturers' protocols.

### Quantitative real time polymerase chain reaction (qRT-PCR)

Total RNA of BMSCs were extracted with Trizol reagent following the manufacturer's instructions and previous studies.^44^ Total RNA (1 μg) was reverse transcribed into cDNA with a Prime Script RT reagent kit (TaKaRa, RR036A). Real-time PCR was performed with TB Green Premix Ex Taq II (Tli RNaseH Plus, TaKaRa, RR420A) using a CFX96 real-time PCR detection system. Data quantification was carried out by a 2^−ΔΔCt^ method, and glyceraldehyde-3-phosphate dehydrogenase (GAPDH) was examined as the reference gene. The primer sequences are listed in Table 1.

**Table 1:** Sequences of primers used in quantitative real-time PCR (qRT-PCR).

Genes	Forward primer	Reverse primer
Gapdh	AACAGCAACTCCCACTCTTC	CCTGTTGCTGTAGCCGTATT
Tigar	GCAGCCAGCATCTTAGTT	CTATGATGAAGACACTGATCCC
Sod1	GTTCCACGTCCATCAGTATG	CCTTTCCAGCAGTCACATT
Cat	ACATGGTCTGGGACTTCT	CCTCTCCATCTGCATTAACC
Gpx1	CAATCAGTTCGGACACCAGAAT	GAGСCTTCTCACCATTCACTTC
Runx2	GCCACTTACCACAGAGCTATT	GAGGCGATCAGAGAACAAACT
Sp7	AAGCCATACGCTGACCTTTC	GGGTGGGTAGTCATTTGCATAG
Alpl	GGATTGACCACGGACATCAT	CCACAGTCAAGGTGTCTTTCT
Sdha	TGTGGACATCAAGACTGGCAAGG	AGGAGCGGATAGCAGGAGGTAC
Actb	CCATCATGAAGTGTGACGTTGAC	CCACCGATCCACACAGAGTACTT

*Gapdh,* glyceraldehyde phosphate dehydrogenase; *Tigar*, p53-induced glycolysis and apoptosis regulator; *Sod1*, superoxide Dismutase 1; *Gpx1*, glutathione peroxidase 1; *Runx2*, runt-related transcription factor 2; *Sp7*, Sp7 transcription factor/osterix; *Alpl*, alkaline phosphatase. *Cat*, Catalase; *Sdha*, succinate dehydrogenase complex flavoprotein subunit A; *Actb*, actin.

### Western blot analysis

Harvested cells were washed in ice-cold PBS and lysed in RIPA lysis. An extraction kit for nuclear proteins (R0050; Solarbio) was used to extract nuclear proteins. Cellular debris was pelleted by centrifugation at 13 000× *g* for 15 min at 4 °C. The protein concentration was determined using the bicinchoninic acid assay. Appropriate amounts of proteins were loaded and separated by SDS-polyacrylamide gel electrophoresis (PAGE), transferred to polyvinylidene fluoride membranes (Millipore, Billerica, MA, USA), and probed with primary antibodies, followed by horseradish peroxidase-conjugated secondary antibody. These are the primary antibodies that were used in this study: anti-mouse TIGAR (1:500, Santa Cruz, sc166290), anti-rabbit Runx2 (1:1000, Cell Signaling Technology, #12556), anti-rabbit sp7 (1:1000, Abcam, ab209484), anti-rabbit Nrf2 (1:1000, Zen-Bio, 380773), anti-rabbit keap1 (1:1000, Zen-Bio, R26935), anti-rabbit p62 (1:1000, Proteintech, 18420-1-AP), anti-rabbit LC3A/B (1:1000, Cell Signaling Technology, #12741), anti-rabbit HDAC1(1:1000, Cell Signaling Technology, #34589), and anti-mouse GAPDH (1:10000, ABclonal, AC002). Secondary antibodies were conjugated with horseradish peroxidase against rabbit or mouse IgG (1:10000, Cell Signaling Technology, 7074 and 7076). The blots were detected using an ECL kit (Bridgen, Beijing, China) through a Photo Documentation and Imaging System (BIO-ID VL, Conn, France), and analyzed by ImageJ software.

### Statistical analysis

Data were analyzed using Prism (version 8.0; GraphPad Software, La Jolla, CA, USA) and all determined parameters are presented as mean ± SEM. ANOVA followed by Tukey's multiple comparison tests was used to compare multiple groups. *p* < 0.05 indicates statistical significance.

## Results

### TIGAR was inhibited in the femur of Dex-induced osteoporosis mice

**Figure 1:**
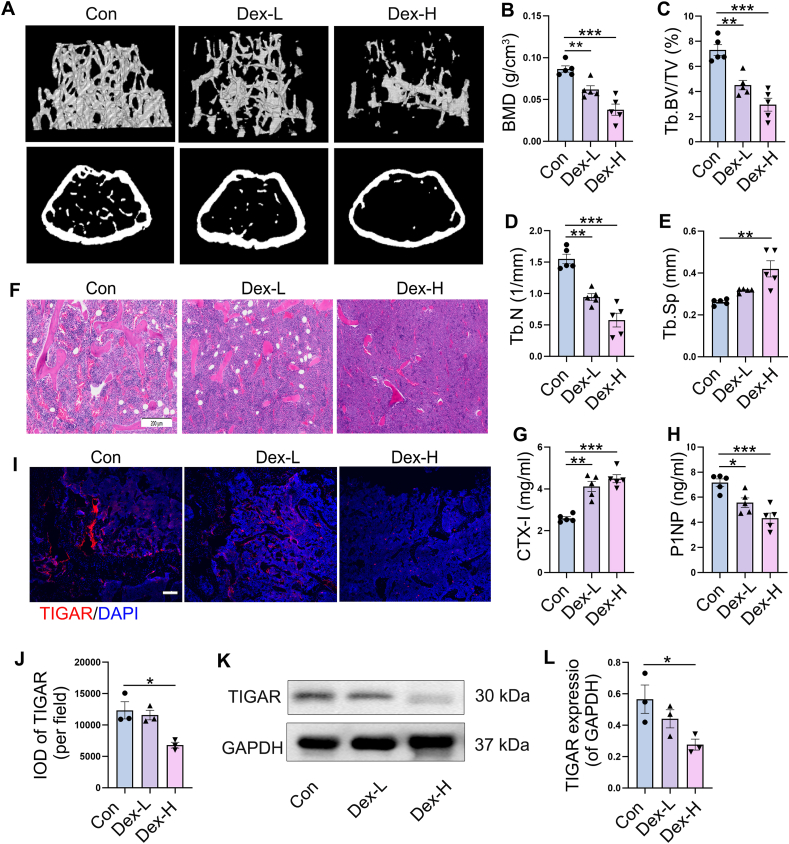
Effects of dexamethasone (Dex) on bone microstructure and TIGAR expression in femur of mice. The mouse model of osteoporosis was established by intramuscular injection of Dex (2 mg/kg and 5 mg/kg) for 8 weeks. **(A)** Representative three-dimensional reconstructive micro-CT images of trabecular bone and cortical bone from the control group and the Dex groups. **(B)** Bone mineral density (BMD), *n* = 5 mice. **(C)** Trabecular bone volume (BV/TV), *n* = 5 mice. **(D)** Trabecular number (Tb.N), *n* = 5 mice. **(E)** Trabecular separation (Tb.Sp), *n* = 5 mice. **(F)** Femur morphological analysis with H&E staining (scale bar, 200 μm). **(G, H)** Level of C-terminal cross linked peptide of type I collagen (CTX-I) (*n* = 5 mice), **(G)** and N-terminal propeptide of type I collagen (PINP) **(H)** in mouse serum. **(I, J)** Confocal microscopy images showing the immunofluorescence staining of TIGAR expression in femur tissue and the quantification of the integrated optical density (IOD) per field. Scale bar, 100 μm; *n* = 3 mice. **(K, L)** Western blot analysis and quantification of TIGAR in femur tissue, *n* = 3. Data are shown as mean ± SEM. Statistical significance was assessed by one-way analysis of variance (ANOVA) with Tukey's multiple comparisons test. ∗∗∗*p* < 0.001,∗∗*p* < 0.01, ∗*p* < 0.05. Dex-L, Dex dose of 2 mg/kg. Dex-H, Dex dose of 5 mg/kg.

The effects of Dex on bone microstructure were observed after continuous injection of Dex into mice for 8 weeks. As compared with the control group, Dex caused a dose-dependent destruction of the bone microstructure and increased bone loss (Fig. 1A), which was emphasized by the quantities of BMD (Fig. 1B), Tb.BV/TV (Fig. 1C), Tb.N (Fig. 1D), and Tb.Sp (Fig. 1E). Similarly, H&E staining revealed sparse trabeculae in the distal femurs of mice treated with Dex (Fig. 1F). In comparison with the control group, Dex treatment increased the level of CTX-I (bone resorption marker) and decreased the level of PINP (bone formation marker) of serum in a dose-dependent manner (Fig. 1G, H). Tissue immunofluorescence and TIGAR immunoblotting were performed to determine the association between TIGAR levels and GIOP. The results showed a degree-dependent decrease in TIGAR expression in the Dex-treated femur compared to that in normal group (Fig. 1I–L). These data suggest that TIGAR expression is inhibited in mice with Dex-induced osteoporosis, indicating a possible correlation between TIGAR and GIOP.

### Dex damaged osteogenesis of BMSCs and decreased TIGAR expression

**Figure 2:**
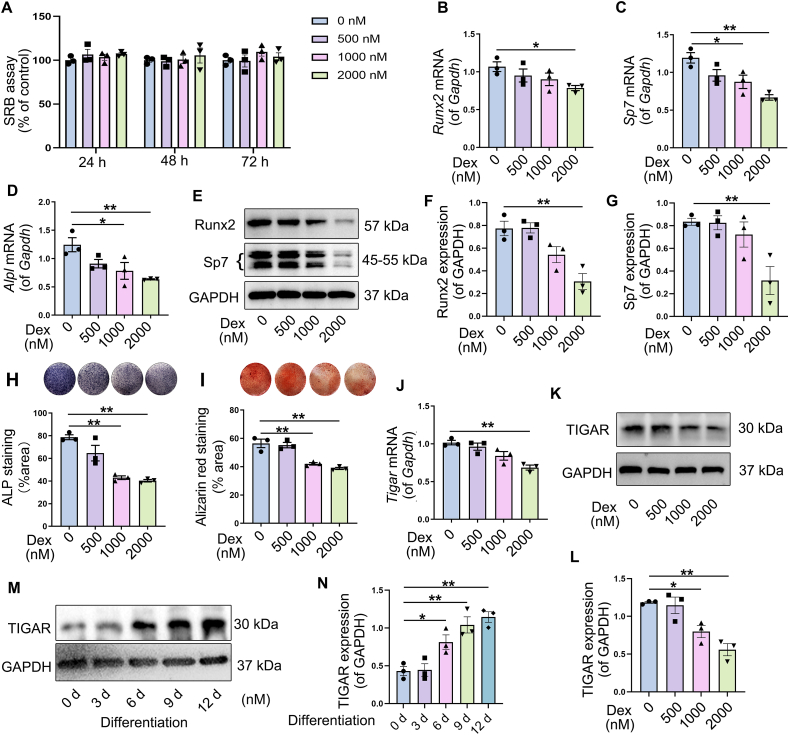
Effects of dexamethasone (Dex) on osteogenic differentiation of bone marrow mesenchymal stem cells (BMSCs) and TIGAR expression. **(A)** BMSCs were treated with Dex for 24, 48, and 72 h, and cells viability was analyzed with sulforhodamine B (SRB) cytotoxicity assay kit. **(B–D)** BMSCs were treated with different concentration of Dex for 48 h, and mRNA levels of runt-related transcription factor 2 *(Runx2)*, Sp7 transcription factor/osterix (*Sp7)*, and alkaline phosphatase (*Alpl)* were detected with qRT-PCR. **(E–G)** Western blotting analysis and quantification of Runx2 and Sp7 in BMSCs treated with different concentration of Dex for 48 h. **(H)** Alkaline phosphatase (ALP) staining and quantification of ALP-positive cells showed osteoblast activity in BMSCs treated with Dex on Day 9. **(I)** Alizarin Red S staining and quantification of mineralized nodules showed the role of Dex on osteogenic differentiation on Day 14. **(J–L)** BMSCs were treated with different concentration of Dex for 48 h; then the expression of TIGAR was detected by qRT-PCR (J) and western blotting (K and L). **(M, N)** Western blot analysis and quantification of TIGAR in osteogenic differentiation at different times (0, 3, 6, 9, and 12 d after addition of osteogenic differentiation medium). Data are shown as mean ± SEM. *n* = 3, biologically independent samples. One-way analysis of variance (ANOVA) with Tukey's multiple comparisons test was used to assess statistical significance. ∗*p* < 0.05, ∗∗*p* < 0.01.

BMSCs were isolated from mice to elucidate the relationship between Dex-induced bone loss and TIGAR expression. We observed the effects of Dex on the viability of BMSCs. The results showed that treatment with different concentration of Dex for 24, 48, and 72 h had no effect on cell viability, respectively (Fig. 2A). The results of qRT-PCR showed that treatment with Dex for 48 h reduced the mRNA levels of genes related to osteogenic differentiation (*Runx2*, *Sp7*, and *Alpl*) in a concentration-dependent manner (Fig. 2B–D). Similarly, the protein expression of Runx2 and Sp7 decreased with increasing Dex concentration (Fig. 2E–G). Alkaline phosphate (ALP) staining revealed that Dex decreased the osteogenic activity of BMSCs in a concentration-dependent manner (Fig. 2H). Alizarin Red S staining also showed a concentration-dependent reduction in calcium nodule formation in BMSCs treated with Dex compared to the control (Fig. 2I). These results revealed the reduced capacity of BMSCs for Dex-induced osteogenic differentiation. Furthermore, the mRNA and protein expression of TIGAR in Dex-treated BMSCs decreased in a concentration-dependent manner (Fig. 2J–L). These results suggested that Dex impedes osteoblast differentiated and decreases TIGAR expression. We also detected TIGAR expression during the osteogenic differentiation of BMSCs. The results showed that TIGAR expression increased from Days 6–12 after osteogenic differentiation, with the highest expression observed on Day 6 (Fig. 2M, N), indicating that TIGAR is involved in osteoblast differentiation.

### TIGAR deficiency was involved in the impairment of osteogenesis of BMSCs under Dex treatment

**Figure 3:**
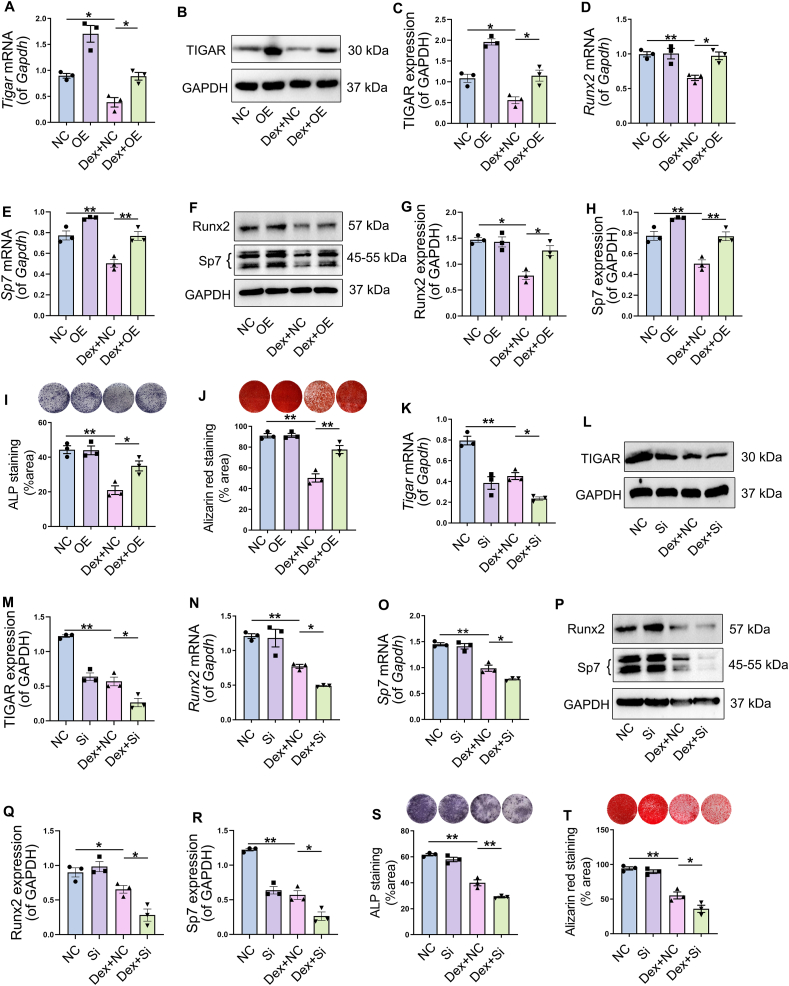
Role of TIGAR on osteogenic differentiation under dexamethasone (Dex) treatment. **(A–C)** Bone marrow mesenchymal stem cells (BMSCs) transfected with TIGAR overexpression plasmid and with or without Dex treatment. TIGAR expression was detected by qRT-PCR (A) and western blotting (B), and (C) is the quantification of TIGAR protein expression. **(D, E)** mRNA expression of runt-related transcription factor 2 *(Runx2)* and Sp7 transcription factor/osterix (*Sp7)* in different treatment groups. **(F–H)** Western blot analysis and quantification of Runx2 and Sp7 in different treatment groups. **(I)** Alkaline phosphatase (ALP) staining and quantification of ALP-positive cells showed osteoblast activity of BMSCs under different treatments. **(J)** Alizarin Red S staining and quantification of mineralized nodules showed the osteogenic differentiation ability of BMSCs under different treatments. **(K–R)** BMSCs transfected with TIGAR siRNA and with or without Dex treatment. Then, TIGAR, Runx2, and Sp7 mRNA and protein expression was detected by qRT-PCR and western blotting. **(S)** ALP staining and quantification of ALP-positive cells under different treatments. **(T)** Alizarin Red S staining and quantification of mineralized nodules under different treatments. Data are shown as mean ± SEM. *n* = 3, biologically independent samples. One-way analysis of variance (ANOVA) with Tukey's multiple comparisons test was used to assess statistical significance. ∗*p* < 0.05, ∗∗*p* < 0.01. NC, negative control. OE, TIGAR overexpression plasmid. Si, TIGAR siRNA.

To explore the role of TIGAR in osteogenesis, we generated cells overexpressing or silencing TIGAR using overexpression plasmids or siRNAs, respectively. First, TIGAR overexpression was performed using TIGAR overexpression plasmid transfection, and the results confirmed that TIGAR mRNA and protein expression decreased by Dex in BMSCs was significantly increased (Fig. 3A–C). Compared to the Dex treatment group alone, TIGAR overexpression upregulated *Runx2* and *Sp7* mRNA expression (Fig. 3D, E), and western blotting also displayed that the decreased expression of Runx2 and Sp7 under Dex was reversed by TIGAR overexpression (Fig. 3F–H). ALP and Alizarin Red S staining showed that the inhibition of osteogenic differentiation induced by Dex was improved by TIGAR overexpression (Fig. 3I, J). TIGAR silence was performed by TIGAR siRNA transfection, and TIGAR expression was confirmed by qRT-PCR and western blotting (Fig. 3K–M). However, the Dex-induced decrease in the mRNA and protein expression of Runx2 and Sp7 was further aggravated when TIGAR was inhibited by siRNA (Fig. 3N–R). Additionally, the results of ALP and Alizarin Red S staining showed that osteogenic differentiation was further suppressed in TIGAR knockdown cells treated with Dex (Fig. 3S, T). Therefore, these results suggest that TIGAR overexpression protects against Dex-induced osteogenic injury, whereas TIGAR knockdown has the opposite effects.

### TIGAR overexpression relieves in Dex-induced osteoporosis in mice

**Figure 4:**
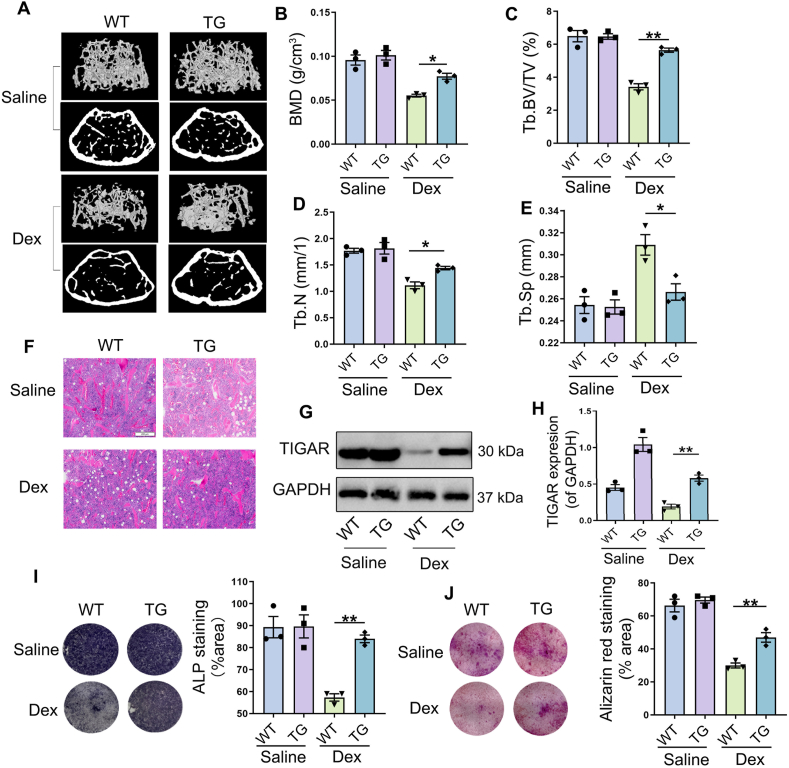
Protective role of TIGAR on dexamethasone (Dex)-induced bone loss. **(A)** Representative three-dimensional reconstructive micro-CT images of the trabecular and cortical bones from the control and Dex groups. **(B)** Bone mineral density (BMD). **(C)** Trabecular bone volume (BV/TV). **(D)** Trabecular number (Tb.N). **(E)** Trabecular separation (Tb.Sp). **(F)** Femur morphological analysis by H&E staining (scale bar, 200 μm). **(G, H)** Western blot analysis and quantification of TIGAR in femur tissue of wild type (WT) and TIGAR transgenic (TG). **(I)** Alkaline phosphatase (ALP) staining and quantification of ALP-positive cells showed osteoblast activity of bone marrow mesenchymal stem cells (BMSCs) derived from WT and TG mice. **(J)** Alizarin Red S staining and quantification of mineralized nodules showed the osteogenic differentiation ability of BMSCs derived from WT and TG mice. Data are shown as mean ± SEM. *n* = 3 mice. Statistical significance was assessed by two-way analysis of variance (ANOVA) with Tukey's multiple-comparisons test. ∗*p* < 0.05, ∗∗*p* < 0.01. WT, wild type; TG, TIGAR transgenic.

The role of TIGAR in Dex-induced osteoporosis was clarified using TIGAR transgenic (TG) mice. Micro-CT and H&E staining showed that in the saline-treated group, compared to WT mice, TG mice showed no significant differences in bone microstructure, BMD, Tb.BV/TV, Tb.N, and Tb.Sp (Fig. 4A–F). Under Dex treatment, compared to the reduced and sparse bone microstructure in WT mice, there was increased bone mass and BMD, elevated Tb.BV/TV and Tb.N, and decreased Tb.Sp in TG mice (Fig. 4A–F). TIGAR expression in the bone tissue was verified by western blotting (Fig. 4G, H). To further verify the effect of TIGAR on osteogenesis, we extracted and cultured BMSCs from the bone marrow of WT, and TG mice to induce osteogenesis and evaluated their osteogenic ability using ALP and Alizarin Red S staining. As the results showed, Dex treatment decreased osteogenic activity and calcium nodule formation in BMSCs derived from WT mice, whereas this phenomenon was alleviated in BMSCs from TG mice (Fig. 4I, J). These results suggest that TIGAR overexpression improves bone quantity and quality by increasing osteogenic differentiation, thus preventing bone loss owing to Dex, which indicating the protective role of TIGAR in GIOP.

### TIGAR prevented Dex-induced defects in osteogenesis through antioxidant action

**Figure 5:**
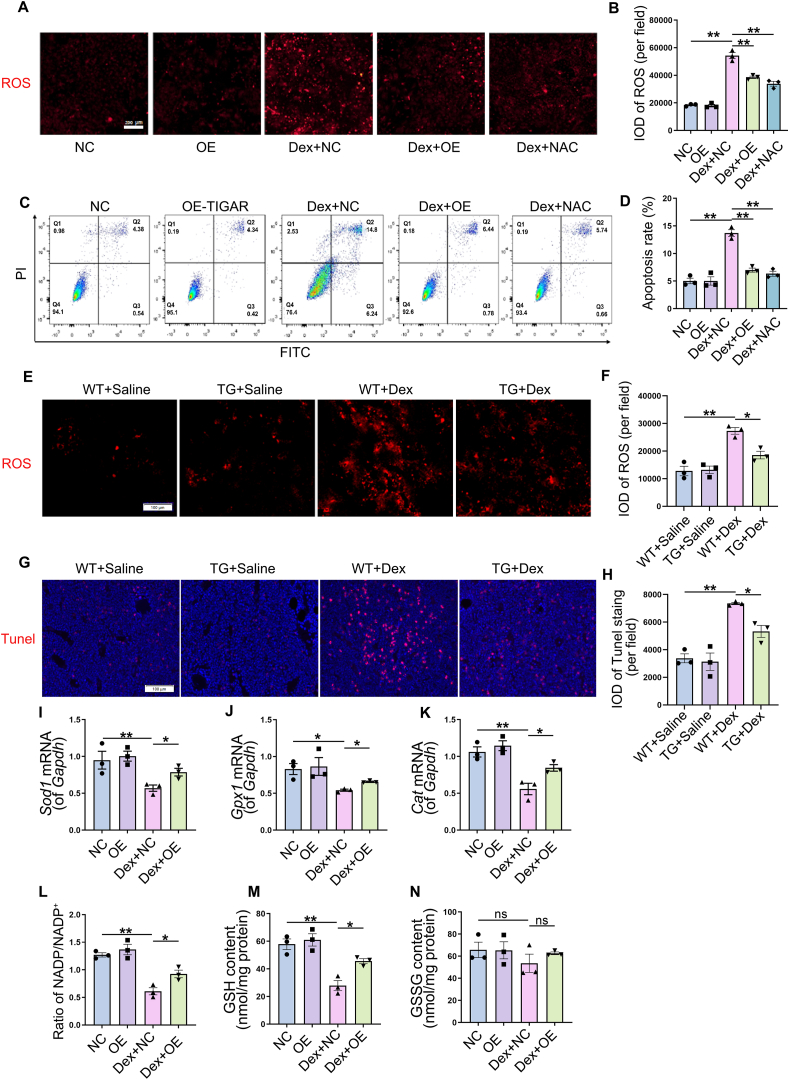
Mechanism of TIGAR preventing dexamethasone (Dex) from inhibiting osteoblastic differentiation. Bone marrow mesenchymal stem cells (BMSCs) were transfected with a TIGAR overexpression plasmid and treated with Dex. N-acetyl-l-cysteine (NAC, 10 mM), an antioxidant, was administrated in cells 1 h prior to Dex treatment. **(A, B)** ROS level was detected by dihydroethidium (DHE) and the quantification of the integrated optical density (IOD) per field. Scale bars, 200 μm. **(C, D)** BMSCs treated with the TIGAR overexpression plasmid and with or without Dex treatment were stained with Annexin-V and PI and detected using flow cytometry, and the apoptosis rate was quantified. **(E, F)** ROS level in femur of WT and TG mice with or without Dex treatment. Scale bars, 100 μm. **(G, H)** Cell apoptosis of femur tissue *in vivo* was detected by Tunel staining and the quantification of IOD per field. Scale bars, 100 μm. **(I–K)** mRNA expression of *Sod1*, *Gpx1*, and *Cat* of cells treated with Dex after TIGAR overexpression. **(L)** Relative NADPH/NADP^+^ ratio. **(M, N)** Glutathione (GSH) and oxidative glutathione (GSSG) contents. *In vitro*: Data are shown as mean ± SEM. n = 3 biologically independent samples. One-way analysis of variance (ANOVA) with Tukey's multiple comparisons test was used to assess statistical significance. ∗*p* < 0.05, ∗∗*p* < 0.01. NC, negative control. OE, TIGAR overexpression plasmid. *In vivo*: data were as the mean ± SEM, n = 3 mice. Statistical significance was assessed by two-way analysis of variance (ANOVA) with Tukey's multiple comparisons test. ∗*p* < 0.05. WT, wild type; TG, TIGAR transgenic.

To investigate the mechanism by which TIGAR protects osteoblast function from Dex-induced inhibition, we evaluated the effects of TIGAR on Dex-induced oxidative damage. The results showed that BMSCs treated with Dex produced significantly more ROS compared to the control, which was downregulated by TIGAR overexpression. And the effect of TIGAR was similar to NAC (ROS scavengers) treatment (Fig. 5A, B). Cell apoptosis was assessed using Annexin-V/PI analysis. According to the results, Dex significantly increased the rate of apoptosis, which was attenuated by NAC treatment or TIGAR overexpression (Fig. 5C, D). We also measured ROS levels and apoptosis in the bone tissue using DHE and Tunel staining *in vivo*. Dex induced ROS production and apoptosis in WT mice, whereas the effect of Dex was relieved in TG mice (Fig. 5E–H). The results suggest that TIGAR overexpression decrease the oxidative stress and apoptosis induced by Dex in osteoblasts.

Finally, expression levels of antioxidant enzymes were analyzed to determine the mechanism underlying the effects of TIGAR on ROS production. The results showed that the mRNA levels of *Sod1*, *Gpx1*, and *Cat* were decreased by Dex treatment, whereas TIGAR overexpression reversed the effects of Dex (Fig. 5I–K). Dex treatment also decreased NADPH/NADP^+^ ratio and glutathione (GSH) content in BMSCs, which was reversed by TIGAR overexpression (Fig. 5L, M). However, there was no significant difference in the amount of oxidized glutathione (GSSG) after Dex treatment (Fig. 5N), suggesting that Dex may decrease the reductive synthesis of GSH by reducing NADPH levels. Taken together, these data suggest that TIGAR prevented Dex-induced defects in osteogenesis by increasing the levels of antioxidant enzymes and NADPH levels to counteract oxidative stress.

### TIGAR reduced Dex-induced oxidative stress through Nrf2

**Figure 6:**
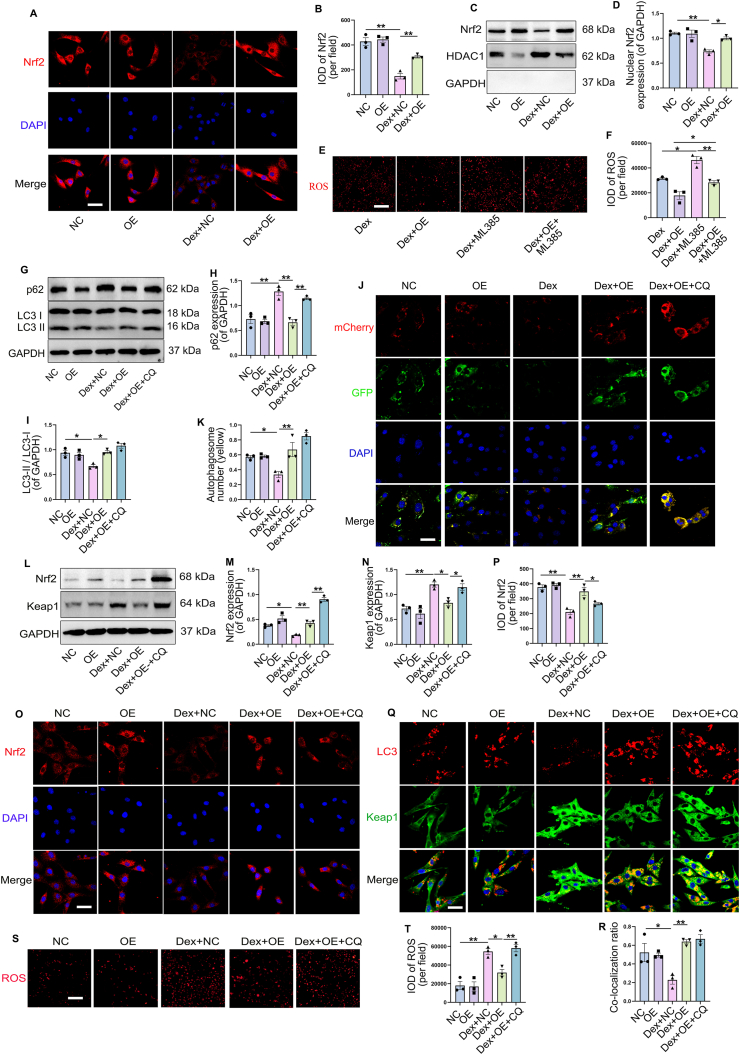
TIGAR activated nuclear factor erythroid-2 related factor (Nrf2) to reduce dexamethasone (Dex)-induced oxidative stress through inducing autophagy. Bone marrow mesenchymal stem cells (BMSCs) were transfected with TIGAR overexpression plasmid and treated with Dex. **(A, B)** Immunofluorescence staining of Nrf2 of BMSCs and the quantification of the integrated optical density (IOD) per field. Scale bars, 50 μm. **(C, D)** Western blot analysis and quantification of Nrf2 expression in extracted nuclear proteins. **(E, F)** ROS level under Dex treatment in BMSCs with or without administration of 10 nM Nrf2 inhibitor (ML385) after transfecting with TIGAR overexpression plasmid, and the quantification of IOD per filed. Scale bars, 100 μm. BMSCs were treated with Dex and chloroquine (CQ) (20 μM) after transfecting with TIGAR overexpression plasmid. **(G–I)** Western blot analysis and quantification of p62 and LC3-II expression under different treatments. **(J, K)** Representative images of mCherry-GFP-LC3 puncta and number of autophagosomes (yellow) (analyzed by Pearson's correlation). Scale bars, 50 μm. **(L–N)** Western blot analysis and quantification of Nrf2 and kelch-associated protein 1 (Keap1) expression under different treatments. **(O, P)** The immunofluorescence staining of Nrf2 in BMSCs and the quantification of the IOD per field. Scale bars, 50 μm. **(Q, R)** Representative immunofluorescence images of LC3 and Keap1. Pearson's correlation of co-localization is shown in the bar graph format from the three independent experiments analyzed. Scale bars, 50 μm. **(S, T)** Representative images of ROS and the quantification of the IOD per field. Scale bars, 100 μm. Data are shown as mean ± SEM. *n* = 3, biologically independent samples. Two-way analysis of variance (ANOVA) with Tukey's multiple comparisons test was used to assess statistical significance. ∗*p* < 0.05, ∗∗*p* < 0.01. NC, negative control. OE, TIGAR overexpression plasmid.

TIGAR can activate Nrf2 to alleviate oxidative stress.^35^ The results showed that Dex decreased the protein expression of Nrf2 as well as its nuclear localization compared with control group, which was rescued by TIGAR overexpression (Fig. 6A, B). Immunoblotting for Nrf2 in the extracted nuclear proteins also confirmed that TIGAR overexpression increased Dex-decreased nuclear Nrf2 expression (Fig. 6C, D). Furthermore, intracellular ROS levels were higher when the Nrf2 inhibitor (ML385) was administered to Dex-treated cells compared to those in the Dex group alone, and ROS mitigated by TIGAR overexpression was also inhibited by the Nrf2 inhibitor in Dex-treated cells (Fig. 6E, F). These results suggested that TIGAR overexpression alleviates Dex-induced oxidative stress by activating Nrf2.

To further explore how TIGAR activates Nrf2, we examined autophagy levels and Keap1 expression in cells. The results showed that compared to the control group, the expression of LC3-II decreased and that of p62 increased with Dex treatment, indicating autophagy inhibition (Fig. 6G–I). However, TIGAR overexpression increased LC3-Ⅱ and decreased p62 expression after Dex treatment, suggesting the activation of autophagy. This was confirmed by chloroquine (CQ, an autophagy inhibitor) treatment, as it further increased LC3-Ⅱ and reversed p62 decline compared with the group treated with Dex + OE (Fig. 6G–I). In the mCherry- GFP-LC3 plasmid transfection assay, the decreased number of autophagosomes (yellow) following Dex treatment was significantly reversed by TIGAR overexpression, whereas CQ administration increased autophagosome accumulation (Fig. 6J, K). These results indicate that TIGAR activates autophagy in BMSCs rather than causing lysosomal dysfunction. We next examined the role of TIGAR-induced autophagy in Nrf2 activation. As shown by immunoblotting, Nrf2 expression, which was upregulated by TIGAR overexpression in Dex-treated cells, was abolished by CQ (Fig. 6L, M). The accumulation of Keap1 following Dex treatment was reduced by TIGAR overexpression, which was abolished by CQ (Fig. 6 L, N). The immunofluorescence results also showed that TIGAR overexpression elevated Nrf2 expression and facilitated the translocation of Nrf2 to the nucleus in Dex-treated BMSCs, which was reversed by CQ (Fig. 6O, P). Further, Keap1 was coated with LC3-labeled autophagy vesicles in TIGAR-overexpressing cells after Dex administration when compared to the Dex group alone, whereas Keap1 was abundant in cells and autophagosomes after inhibition of autolysosome degradation by CQ administration (Fig. 6Q, R). These results suggest that autophagy is necessary for TIGAR to activate Nrf2. In addition, TIGAR-induced antioxidant activity in Dex-treated BMSCs was abolished by the administration of CQ, which was indicated by the reversed fluorescence of DHE (Fig. 6S, T). Collectively, these data demonstrated that TIGAR induced autophagy to degrade Keap1, and thus activated Nrf2 to play an antioxidant role in BMSCs treated with Dex.

## Discussion

GIOP is a secondary osteoporosis associated with high risk of fracture and heavy economic burden, and there is still a large treatment gap although several therapeutic drugs are currently available to treat GIOP.^45^ It is necessary to further elucidate the mechanism of GIOP and explore novel therapeutic strategies. TIGAR plays an important role in diseases such as cancer, stroke, Alzheimer's disease, Parkinson's disease, chronic lung inflammation, heart failure, and myocardial ischemia,^25^^,^^35^^,^^39^^,^^46^ and the recent studies indicated that TIGAR may be a viable therapeutic strategy for obesity.^47^ However, TIGAR's role in GIOP has not been previously reported. In the current study, unlike other stress conditions that usually lead to the elevation of TIGAR expression,^26^^,^^39^^,^^48^ we found Dex decreases TIGAR expression in bone tissue and pre-osteoblasts, which may be related to the long-term administration of Dex or the different response of TIGAR to stress in different tissues or cells. These results indicates that TIGAR may be associated with GIOP. Previous studies on the progression of dementia suggested that reduced TIGAR expression may have a negative impact on cell survival.^49^ Furthermore, we demonstrate that TIGAR overexpression improves Dex-induced osteoporosis by promoting osteogenic differentiation and activity *in vitro* and *in vivo*. Taken together, the current studies indicated that Dex damaged TIGAR expression in bone tissues and pre-osteoblasts, whereas restoration of TIGAR expression can alleviate Dex-induced osteoporosis in mice by improving osteogenic activity.

Bone formation depends on the number and activity of osteoblasts after differentiation. Dex impairs osteogenic differentiation and inhibits osteogenic formation by inducing excessive oxidative stress and subsequent apoptosis, which is the main mechanism of GIOP.^50^ TIGAR promotes stem cell differentiation by regulating associated transcriptional activators and repressors, and prevents cell injury from oxidative stress and apoptosis by promoting the production of NADPH, which is important for maintaining reduced GSH levels and antioxidant system.^25^^,^^51^^,^^52^ Additionally, findings from another study in our group demonstrated that in embryonic bone tissues, TIGAR exhibited stronger co-localization with the osteoblast marker Runx2 compared to its co-localization with the osteoclast marker TRAP (Fig. S2), which indicated that TIGAR likely exerts its protective role against Dex-induced osteoporosis primarily through actions in osteoblasts. However, whether TIGAR alleviates GIOP by reducing the intracellular ROS levels in osteoblasts remains unclear. Similar to previous studies,^53^, ^54^, ^55^ in the current study, we found that overexpression of TIGAR significantly decreased Dex-induced ROS production and the apoptosis rate in Dex-treated BMSCs and mouse femur. Further, TIGAR overexpression also significantly reversed the reduction of antioxidant enzymes (*Sod1*, *Gpx1* and *Cat*) expression, NADPH/NADP^+^ ratio and GSH content in Dex-treated BMSCs. We also examined TIGAR expression in osteoblasts (MC3T3E1), mature osteocytes (MLOY4) or osteoclasts (differentiated from bone marrow derived macrophage (BMDM)) following Dex treatment, and observed the antioxidant effect of TIGAR overexpression in these cell types. The results of MC3T3E1 were consistent with those of the pre-osteoblast (BMSCs) (Fig. S3). However, in MLOY4 cells, Dex treatment did not significantly alter TIGAR expression, yet TIGAR overexpression still mitigated Dex-induced ROS increase and antioxidant enzyme expression decrease (Fig. S4). Similarly, in BMDM-derived osteoclasts, Dex treatment had no obvious effect on TIGAR expression, and TIGAR overexpression had no obvious effect on the accumulation of ROS and the reduction of antioxidant enzyme expression triggered by Dex (Fig. S5). These results suggest that TIGAR mainly acts on osteoblasts and reduces intracellular ROS levels and osteoblast apoptosis by rescuing the antioxidant system and promoting production of NADPH, thereby promoting the survival and function of osteoblasts and increasing bone formation.

TIGAR has been reported to activate Nrf2 by inducing autophagy to degrade keap1 in neurons with prolonged ischemia.^35^ Our results showed that Dex decreased Nrf2 expression, and TIGAR overexpression rescued the Dex-induced reduction in cytoplasmic and nuclear Nrf2 expression. Nrf2 inhibitors abolished TIGAR-reduced ROS production in Dex-treated osteoblasts, suggesting that Nrf2 activation is required for the antioxidant activity of TIGAR. The potential mechanism of Nrf2 downregulation by Dex may also be: (1) Dex binds to glucocorticoid receptors to inhibit downstream gene transcription. (2) Keap1 binds to Nrf2 to induce ubiquitination and degradation of Nrf2, and Dex may promote the degradation of Nrf2 by increasing Keap1 accumulation.^56^ (3) Dex may induce ubiquitination degradation of Nrf2 through miRNA.^57^ Therefore, the activation of Nrf2 by TIGAR may involve several regulatory mechanisms in Dex treated osteoblasts.

We suspected that TIGAR decreases oxidative stress through the autophagic degradation of keap1 under Dex treatment. Consequently, we examined LC3-Ⅱ and p62 expression reflecting autophagy flux by immunoblotting. Previous studies have shown that TIGAR inhibits autophagy.^24^^,^^39^^,^^52^ In our study, we found that when Dex was administered at high concentrations for 48 h, TIGAR overexpression significantly reversed Dex-induced autophagy inhibition, which indicated that TIGAR may activate autophagy in response to intense stress or injury, thus alleviating cell damage. Furthermore, we demonstrated that TIGAR activates Nrf2 by inducing autophagy degradation of Keap1 by using the autophagy inhibitor chloroquine. Taken together, these results suggest that TIGAR overexpression activates autophagy and thus degrades keap1 in Dex-treated pre-osteoblast, which promotes Nrf2 nuclear translocation and activation, thus reducing intracellular oxidative stress.

## Limitations and conclusions

The research on TIGAR in GIOP has not been reported previously. The current study indicates that TIGAR overexpression activates Nrf2 to reduce intracellular oxidative stress under Dex treatment by inducing autophagy to degrade Keap1, thus alleviating GIOP. However, there are some limitations in our study. For example, the dynamic changes of TIGAR and its regulation in GIOP progress need to investigate to clarify the protective role of TIGAR in further studies. Although TIGAR as a target to treat diseases (such as nervous system diseases, cardiovascular diseases, etc.) has great application potential,^25^^,^^58^ so far there are no TIGAR agonists and the clinical application of TIGAR overexpression remains to be explored, as it is closely associated with tumorigenesis.^59^ In addition, how Dex regulates TIGAR expression is not evaluated in this study. TIGAR expression can be regulated in different types of cells by the p53 family, and some transcription factors, such as SP1 and CREB, and non-coding miRNAs, and the histone methyltransferase family and Wnt signaling pathway, etc.^25^ It has been reported that inhibition of p53 activation can relieve the inhibition of osteogenic differentiation, thus alleviating Dex-inhibited osteogenic formation.^60^^,^^61^ Although it might be expected that the role and expression of TIGAR are independent of P53 in our study, the exact mechanism needs to be explored further in future studies.

In summary, our study revealed that TIGAR may alleviate GIOP by activating autophagy-Nrf2 signaling to reduce oxidative stress and apoptosis in osteoblasts, suggesting that TIGAR may serve as a viable therapeutic strategy for GIOP.

## CRediT authorship contribution statement

**Dingmei Zhang:** Writing – original draft, Methodology, Funding acquisition. **Feng Ding:** Writing – original draft, Investigation, Formal analysis. **Yizhong Wang:** Software, Investigation. **Jie Cheng:** Writing – original draft, Methodology. **Jiaxing Zhu:** Validation, Data curation. **Shiyu Liu:** Writing – review & editing, Funding acquisition. **Xin Wang:** Writing – review & editing, Conceptualization. **Zheng-Hong Qin:** Writing – review & editing, Project administration, Conceptualization. **Lili Ren:** Writing – review & editing, Supervision, Conceptualization.

## Funding

This work was funded by National Natural Science Foundation of China (No. 82260440), Guizhou Science and Technology Fund Project (Qian Jiao Ke He Jichu-ZK[2023]-yiban 584), Innovative Talent Project of Shaanxi province (2020KJXX-057), Educational Commission of Guizhou Province (Qian Jiao He KY Zi 2022-283), The Doctoral Scientific Research Foundation for Affiliated Hospital of Zunyi Medical University (No. 2022-5), Collaborative Innovation Center of Chinese Ministry of Education (No. 2020-39), the Science and Technology Fund Project of Guizhou Provincial Health Commission (No. gzwkj 2022-108), Medical Research Union Found for High-quality health development of Guizhou Province (2024GZYXKYJJXM0138).

## Conflict of interests

The authors declare no potential competing interests.
