# Supplementary material for: TIGAR promotes osteogenic differentiation and ameliorates glucocorticoid-induced osteoporosis via autophagy-Nrf2-ROS axis
Source: Genes Dis. 2025 Jun 26;13(2):101735. doi: 10.1016/j.gendis.2025.101735 (PMC12757533; doi:10.1016/j.gendis.2025.101735)
Supplement: Multimedia component 1 [file mmc1.docx]

**Supplementary Figures:**


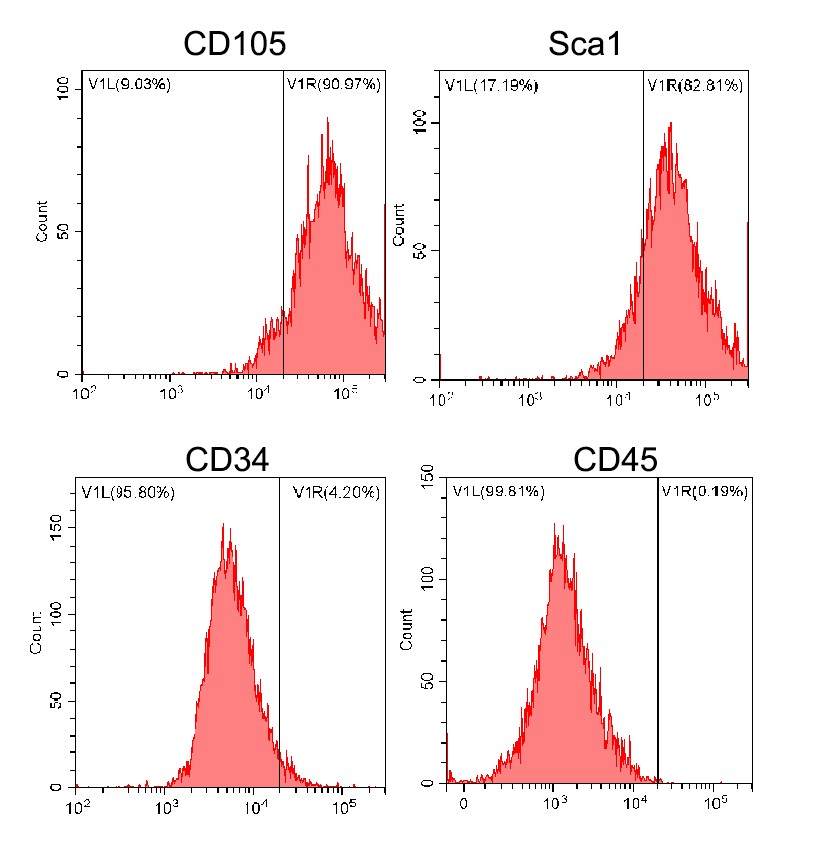


Figure S1. The positive surface markers CD105 and Sca1 and negative marker CD34 and CD45of BMSCs were analyzed by flow cytometry.


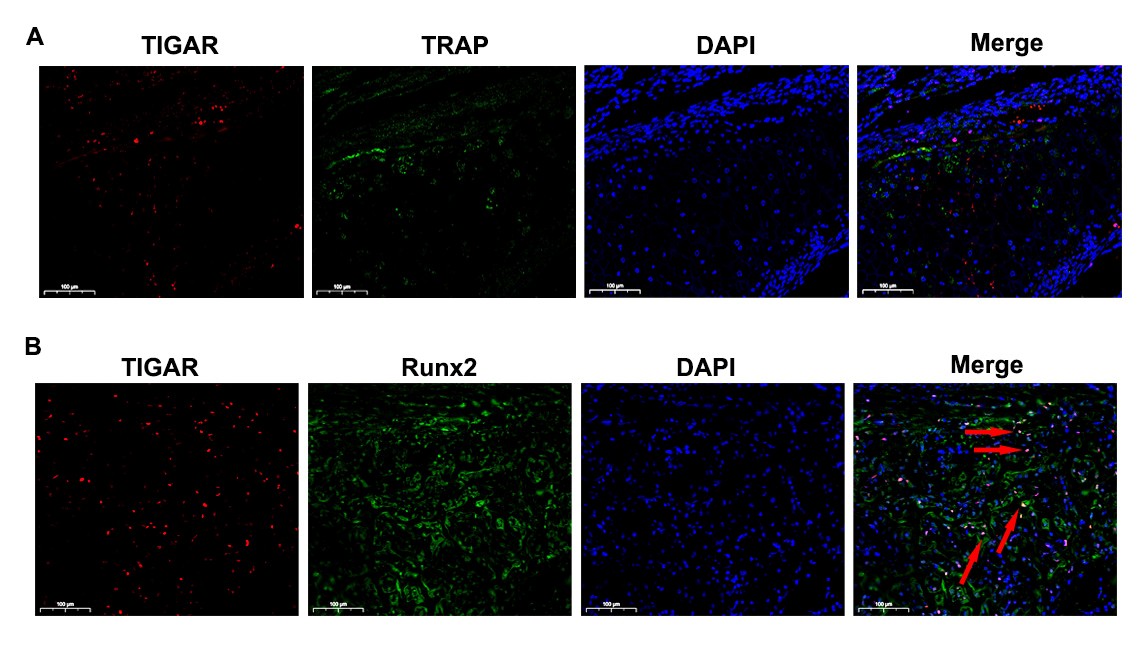


Figure S2. TIGAR exhibited stronger co-localization with the osteoblast marker Runx2 compared to its co-localization with the osteoclast marker TRAP. A. TIGAR (red) co-localization with the osteoclast marker TRAP (green). B. TIGAR (red) co-localization with the osteoblast marker Runx2(green). Scale bars, 100 μm.

**
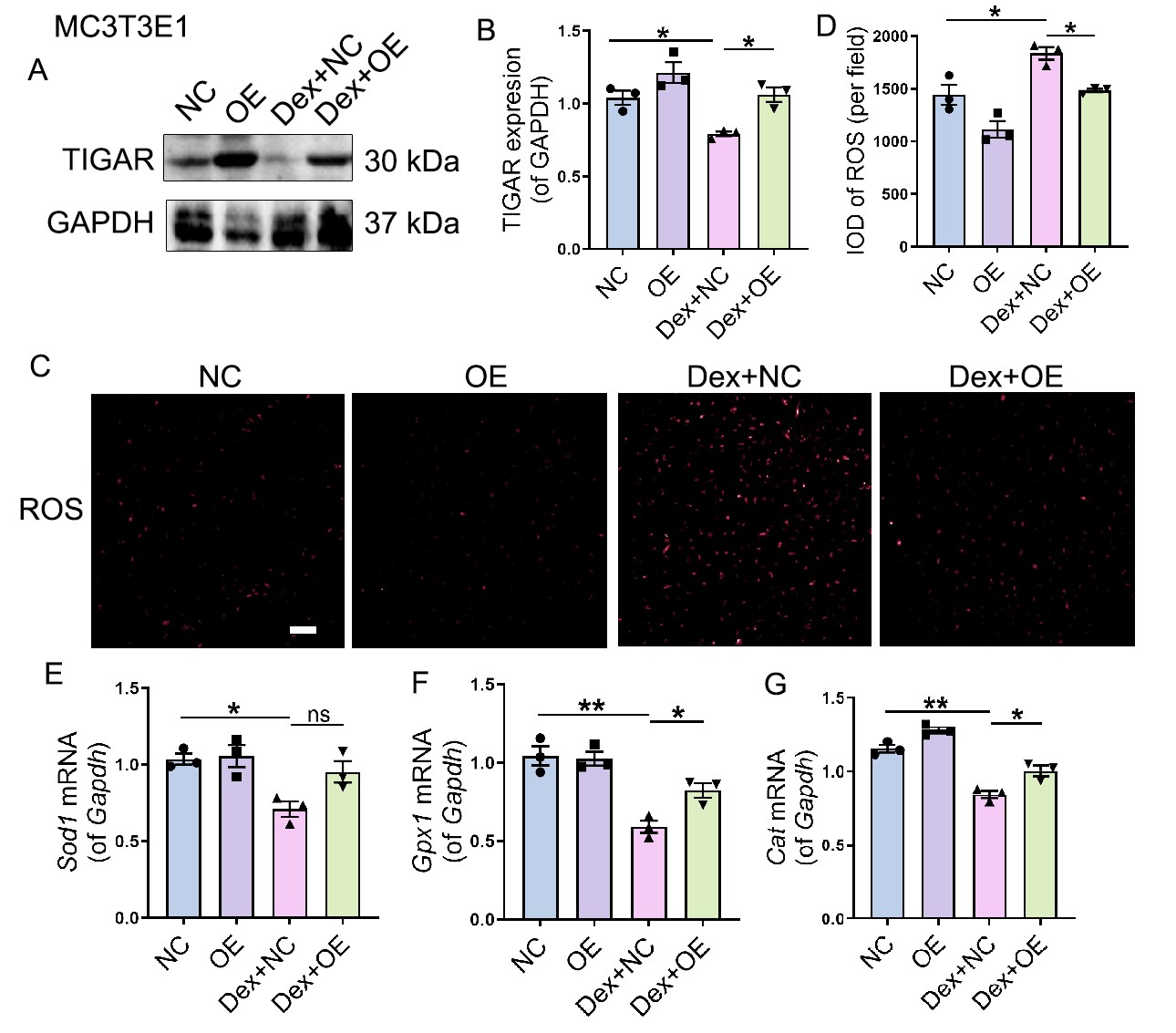
**

Figure S3. The expression and antioxidant effect of TIGAR in pre-osteoblasts (MC3T3E1). MC3T3E1 transfected with TIGAR-overexpressing plasmid (OE) and with or without 2 μM Dex treatment for 48 h. A, B. Western blotting analysis and quantification of TIGAR. C, D. ROS level was detected by dihydroethidium (DHE) and the quantification of the integrated optical density (IOD) per field. Scale bars, 200 μm. E-G. mRNA expression of *Sod1*, *Gpx1*, and *Cat*. Data are shown as mean ± SEM. n= 3 biologically independent samples. One-way analysis of variance (ANOVA) with Tukey’s multiple comparisons test was used to assess statistical significance. **P* < 0.05, ***P* < 0.01. NC, negative control. OE, TIGAR overexpression plasmid.


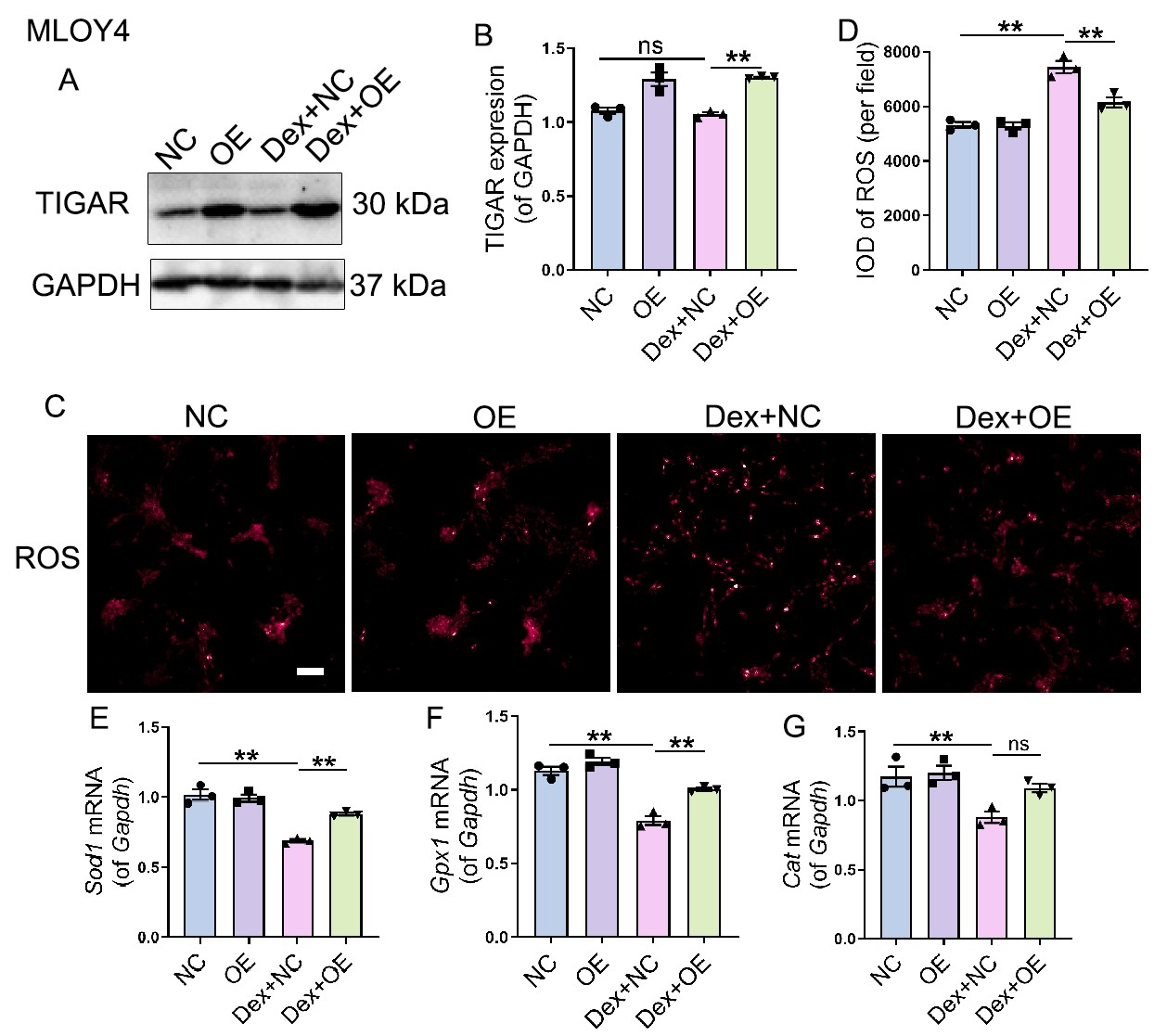


Figure S4. The expression and antioxidant effect of TIGAR in mature osteocytes (MLOY4). MLOY4 transfected with TIGAR-overexpressing plasmid (OE) and with or without 2 μM Dex treatment for 48 h. A, B. Western blotting analysis and quantification of TIGAR. C, D. ROS level was detected by dihydroethidium (DHE) and the quantification of the integrated optical density (IOD) per field. Scale bars, 200 μm. E-G. mRNA expression of *Sod1*, *Gpx1*, and *Cat*. Data are shown as mean ± SEM. n= 3 biologically independent samples. One-way analysis of variance (ANOVA) with Tukey’s multiple comparisons test was used to assess statistical significance. **P* < 0.05, ***P* < 0.01. NC, negative control. OE, TIGAR overexpression plasmid.


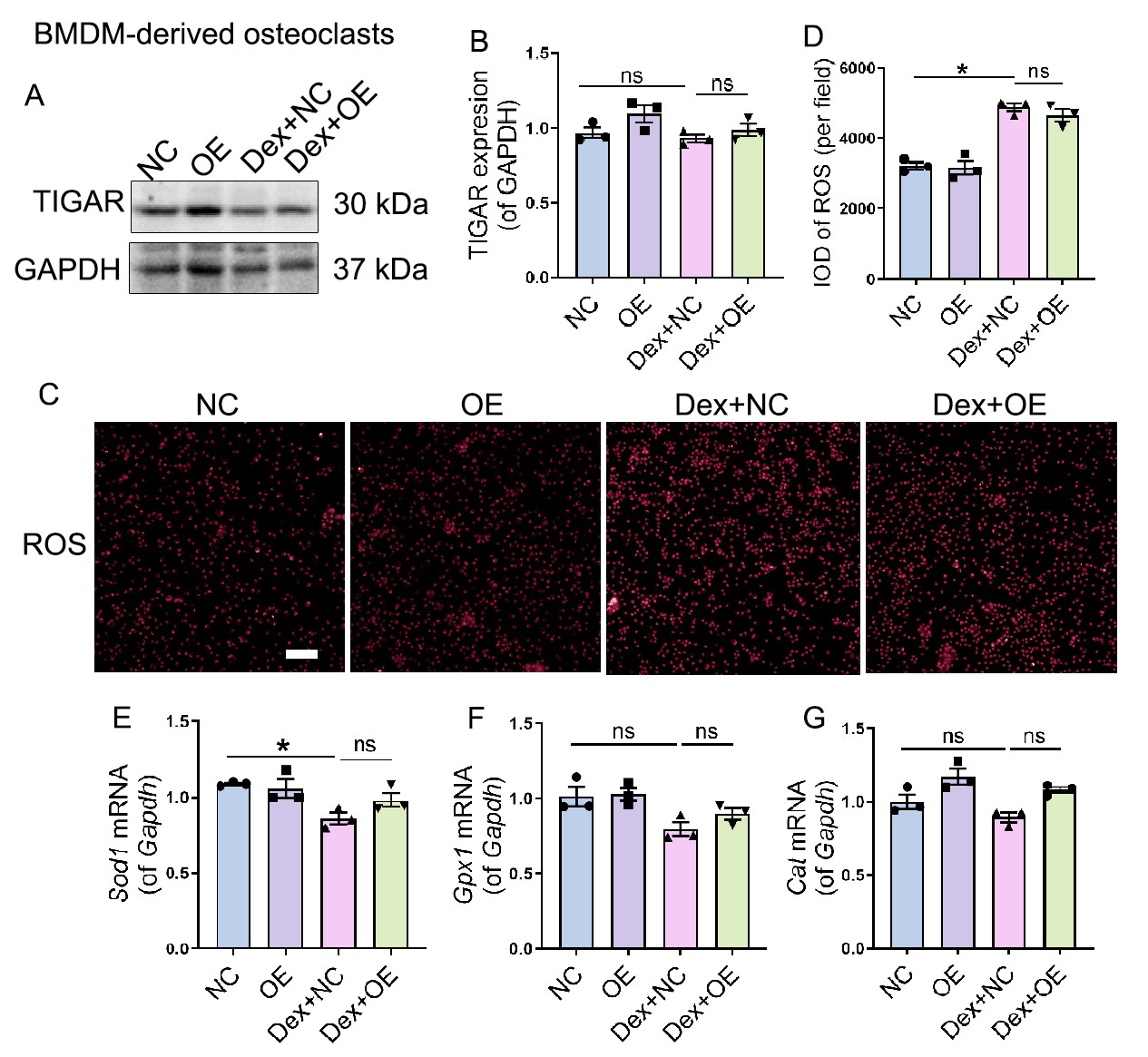


Figure S5. The expression and antioxidant effect of TIGAR in bone marrow derived macrophage (BMDM)-derived osteoclasts. After stimulating BMDMs with RANK ligand (RANKL) to induce osteoclast differentiation, cells were transfected with TIGAR-overexpressing plasmid (OE), followed by 2 μM Dex treatment for 48 h. A, B. Western blotting analysis and quantification of TIGAR. C, D. ROS level was detected by dihydroethidium (DHE) and the quantification of the integrated optical density (IOD) per field. Scale bars, 100 μm. E-G. mRNA expression of *Sod1*, *Gpx1*, and *Cat*. Data are shown as mean ± SEM. n= 3 biologically independent samples. One-way analysis of variance (ANOVA) with Tukey’s multiple comparisons test was used to assess statistical significance. **P* < 0.05, ***P* < 0.01. NC, negative control. OE, TIGAR overexpression plasmid.
